# Using field training in indigenous communities as a method of creating awareness of the one health concept among Malaysian university students: a non-experimental pre and post-test intervention study

**DOI:** 10.1186/s42522-020-00023-6

**Published:** 2020-09-03

**Authors:** Abdul Rashid, Seng Fong Lau

**Affiliations:** 1 Department of Public Health Medicine, RCSI&UCD Malaysia Campus, 4 Sepoy Lines, George Town, 10450 Penang, Malaysia; 2 Department of Veterinary Clinical Studies, Faculty of Veterinary Medicine, University Putra Malaysia, Serdang, Malaysia

**Keywords:** One health, *Orang Asli*, Indigenous, Field training, Multidisciplinary

## Abstract

**Background:**

This paper describes the result of workshops conducted to increase the knowledge and awareness of university students using a multidisciplinary, collaborative, multisectoral and trans-disciplinary approach concerning One Health and the indigenous people of peninsular Malaysia called the *Orang Asli*.

**Methods:**

A non-experimental pre and post-test intervention study was carried out among medical, veterinary and allied health students from six public and private universities who attended workshops on One Heath in two *Orang Asli* communities living by the Temenggor lake in Malaysia as part of the Malaysia One Health University Network (MYOHUN) efforts in training future and present One Health workforce.

**Results:**

There was a significant increase in various aspects of knowledge and interest concerning One Health and the *Orang Asli*. The mean knowledge scores of One Health (p < 0.001) and *Orang Asli* (p < 0.001) increased significantly post workshop. A repeated measures ANOVA with a Greenhouse-Geisser correction showed the mean scores of knowledge of One Health F (1, 166) = 127.198, p < 0.001) and *Orang Asli* F (1, 166) = 214.757, p < 0.001) differed statistically significantly between the two time points. The test revealed that the score differences for knowledge on One Health (mean difference = 1.796, p < 0.001) and *Orang Asli* (mean difference = 4.940, p < 0.001) were statistically significant. Repeated measures ANOVA showed a significant difference in the knowledge on *Orang Asli* between the students of different courses F (4,166) = 3.734, p-0.006. The difference in the One Health knowledge scores between the students of different courses was not statistically significant F (4,166) = 0.998, p = 0.410.

**Conclusions:**

Emphasis on field training in relation to One health can provide university students greater levels of preparedness to combat zoonotic diseases.

## Background

It is estimated that there are approximately 370 million indigenous individuals, representing more than 5000 distinct peoples living in more than 90 countries [1]. These indigenous populations make up one third of the world’s poor and their territories overlap all biodiverse regions of the world [2–4]. *Orang Asli*, the indigenous people of peninsular Malaysia, are ethnic minorities having their own unique languages, knowledge, and beliefs. They live close to tropical forests, adapting to forest ecosystems which is reflected by their traditional lifestyle. The flora and fauna collected from the tropical forest, is the main source of income for these communities besides the daily food and seasonal bounties of the forest [5].

Approximately 75% of human emerging infectious diseases are caused by zoonotic diseases, which may result in high fatality rates [6]. Studies have shown that the prevalence of zoonotic diseases are disturbingly high among indigenous peoples worldwide, including Malaysia [7–10]. In comparison to the general population, the *Orang Asli* have lower health status [11] and are at higher risk of illnesses especially zoonotic infections because of their low levels of education, poor hygienic practices and bush meat eating habits [6–8]. Because of poverty, most do not consider buying soap for hand washing as a necessity. They have poor access to social and health care services because most of them live in remote locations. All these factors culminate to the *Orang Asli* having higher mortality rates in comparison to non-indigenous groups and overall shorter life expectancy due to high burden of diseases [12, 13].

With the complex pattern of emergence and re-emergence of infectious diseases, separated sectoral thinking delays the process of combating these illnesses. Ideas that go beyond just human and animal diseases have been implemented in the One Health concept. The One Health concept is defined as expanding interdisciplinary collaborations and communications in all aspects of health care for people, animals and the environment [14–16]. This transdisciplinary One Health approach integrates frameworks and methodologies beyond traditional classroom settings and involves multi-disciplines including policy makers and communities, which are prerequisites to determine healthy lifestyles of human beings, by providing an interconnection of human, animal and their social and ecological environment at global, national and local levels [17].

Multidisciplinary approach enables students from different universities to interact and develop critical thinking and hone their skills. Using the One Health concept, academic institutions can play a foundational role in breaking down silos and facilitating transdisciplinary research initiatives. Field training programmes can make an impact in the understanding of the culture and living lifestyle of *Orang Asli,* which is critical in improving the health status of the *Orang Asli,* by education and by creating awareness towards zoonotic diseases, resulting in change in attitudes and improvement of hygiene practices [18].

Studies have shown that field training programmes alongside classroom teaching are successful in getting participants to detect and respond effectively to infectious disease outbreaks, improve the students ability to diagnose, treat and prevent infectious diseases and make students appreciate the real life challenges and issues arising in the field such as limited resources and facilities, besides having fun learning [19–21]. With this in mind and in response to the growing zoonotic disease threats among the *Orang Asli*, several workshops and field training sessions were organized in the *Orang Asli* communities living by the Temenggor lake in Perak, Malaysia to train present and future One Health workforce. These workshops were organized by Malaysia One Health University Network (MYOHUN) to create awareness and to enhance knowledge of students in different disciplines from Malaysian Universities concerning One Health and the *Orang Asli* communities. Pre- and post-workshops assessments were conducted to evaluate the outcomes of the workshops and field training. The outcomes of the assessment can be used as a guideline for future training of One Health workforce at higher education institutions. This paper describes the success of the workshops in increasing the knowledge and awareness of One Health and of the *Orang Asli.*

## Methods

### Study design

This is a non-experimental pre and post-test intervention study.

### Setting and sampling

This study was carried out among medical, veterinary and allied health students from six public and private universities who attended workshops on One Heath in two *Orang Asli* communities living by the Temenggor lake in Perak, one of the 13 states in Malaysia. The *Orang Asli* or the indigenous people of peninsular Malaysia comprise of eighteen official tribes under three main ethno-linguistic groups, Negrito, Senoi and Proto-Malay. Each sub-ethnic group has its own language and culture and have varied occupations and ways of life.

This study was carried out in the *Jahai* and *Temiar Orang Asli* settlements in Sungai Tiang village and Pos Kemar located in the Belum-Temenggor Forest. This study was conducted in these remote *Orang Asli* villages which are without electricity and treated water although parts of Pos Kemar has been recently electrified. The *Jahai* villagers are mostly hunter gatherers while the *Temiar* villagers are a mixture of hunter gatherers and farmers. The villagers from both the sites are at risk of zoonotic infections due to their low levels of education and poor hygienic practices and bush meat-eating habits. While the *Temiar* village has a health clinic, the *Jahai* villagers only have access to the closest health centre by an hour’s boat ride to the jetty followed by 40 min’ drive. This village is visited by a mobile health team from the Ministry of Health Malaysia, usually headed by a nurse whose focus is maternal and child health. There were approximately 437 villagers making up 102 families in the *Jahai* village whereas there were approximately 8000 villagers in the *Temiar* settlement. Because of poverty, most do not consider buying soap for hand washing as a necessity. This field training was part of the Malaysia One Health University Network (MYOHUN) initiative to train current and future One Health workforce by creating awareness and enhancing their knowledge on One Health.

### Tools

A questionnaire was especially designed for this study. Besides baseline information which included age, institution and gender, there were questions related to knowledge which included how much they knew and interacted with the *Orang Asli*, the *Orang Asli’s* access to treated water and hygienic practices, bush meat related knowledge and interests in knowing more about the *Orang Asli*. Questions related to One Health included knowledge on who could be involved in a One Health team, the most common human pathogen, emerging zoonosis and their origins and if forest degradation helps spread of zoonotic infections. Students self-rated, on a scale of 1 to 5 where 1 is very poor and 5 very good, concerning one health knowledge, their interest in one health, interest in learning more about One Health and interest in working in a one Health team. The questionnaire was administered on the first day of the workshop and re-administered on the third and final day.

### Workshop content

All the students stayed on a boat house during the period of the workshop. On the first day they were given lectures regarding One Health, *Orang Asli* and zoonotic diseases. They were required to prepare a health promotion and education programme for the *Orang Asli* children using a multidisciplinary approach to enhance the knowledge of the *Orang Asli* children concerning zoonotic infections. To inculcate multidisciplinary teamwork, they were divided into small groups comprising of students from different institutions and different courses. The students were supervised by trained multidisciplinary facilitators from six different institutions. On the second day they were taken into the *Orang Asli* village to conduct the programme and for on-site teaching on zoonotic infections, One Health and the *Orang Asli*. In the evening of the second day they were involved with discussions and reflections relating to their activities. On the final day morning they were taken to a salt lick to discuss wildlife habitat and ecology. The participants were also trained on interpersonal and communication skills. A post intervention survey was conducted on the last day. This workshop was done in six batches of approximately 30 students in each batch over a period of 2 years. The time period spent on each location was equal.

### Analysis

Data was analysed using PASW version 18. The proportion of each answer was compared using Mc Nemar’s test for statistical significance. Likert scale questions were analysed using Wilcoxon’s test. The pre and post mean scores of the students’ knowledge on *Orang Asli* and One Health were analysed using paired t test. Repeated measures ANOVA was conducted to compare the effects of different student courses on One Health and *Orang Asli* knowledge. A probability value of P < 0.05 was considered to be significant.

### Ethics

The research was conducted ethically. All respondents were read the participant information sheet which contained the information concerning the study including the rights of the participant to refuse and exit from the study at any moment. Each participant was asked for an informed verbal consent before proceeding with the questionnaire. The study received approval from the Joint Penang Independent Ethics Committee (PMC ID: 201803–01 RC8). The anonymity of the respondents is assured.

## Results

In total there were 171 students from six institutions pursuing different courses, 42 doing Medicine (24.6%), six microbiology (3.5%), 56 veterinary (32.7%), 56 allied heath (32.7%) and 11 Public Health (6.4%). Majority were female (73.7%) and the mean age of the participants was 23 (range 18 to 37).

**Fig. 1:**
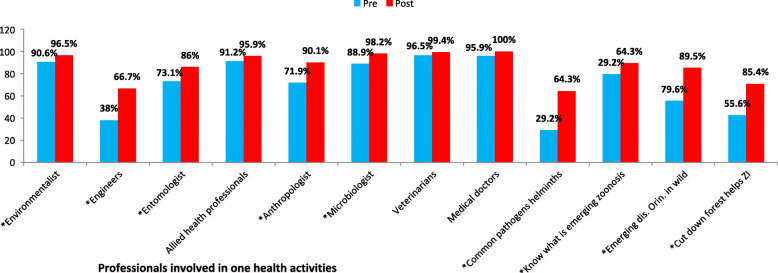
One Health knowledge

As shown in Fig. 1, in general there was an increase in the knowledge of the participants relating to One Health post workshop. The increase in the correct answers post workshop for the most common pathogen (p < 0.001), awareness of emerging zoonosis (p < 0.001) and its common origin (< 0.001) and how deforestation can spread zoonotic infections (p < 0.001) were statistically significant. The increase in correct answers post workshop on the professions involved in One Health activity for microbiologist (< 0.001), anthropologist (< 0.001), entomologist (< 0.001), engineers (< 0.001) and environmentalist (< 0.001) were statistically significant.

**Fig. 2:**
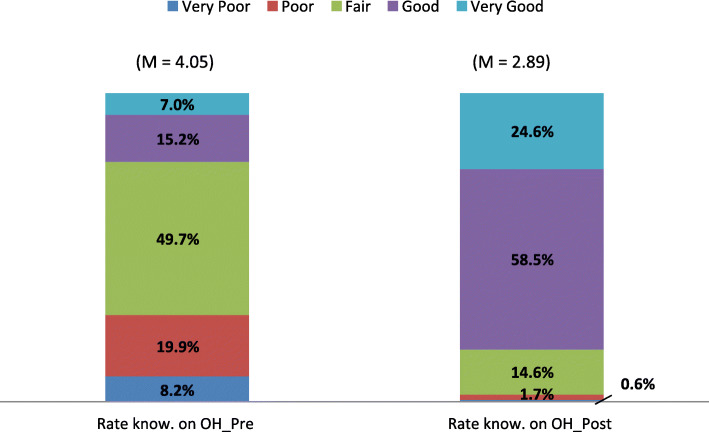
Self-rated knowledge related to One Health

Self-rated knowledge concerning One Health improved post workshop and this change was statistically significant (p = 0.001) (Fig. 2).

**Fig. 3:**
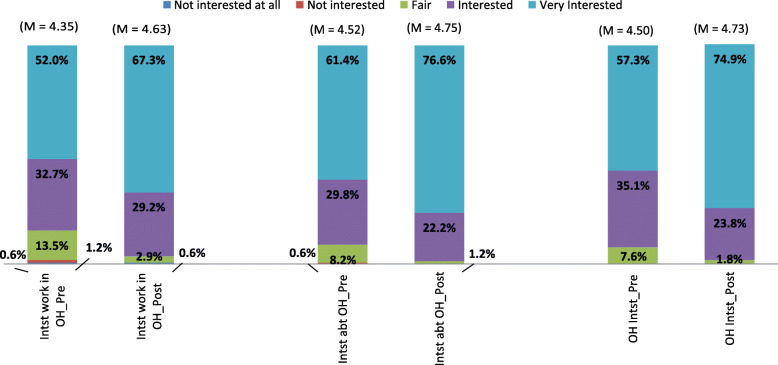
Interest in One Health

Figure 3 depicts the interest shown in One Health, which improved post workshop. The increase in interest (< 0.001), learning more (< 0.001) and working in a One Health team (< 0.001) was statistically significant.

Table 1 shows the mean knowledge score increased post workshop; this difference was statistically significant (p < 0.001).

**Table 1:** Mean difference in One Health knowledge

One Health knowledge	Mean	Paired t test	Significance
Pre Post	8.43 10.43	−12.309	< 0.001

**Fig. 4:**
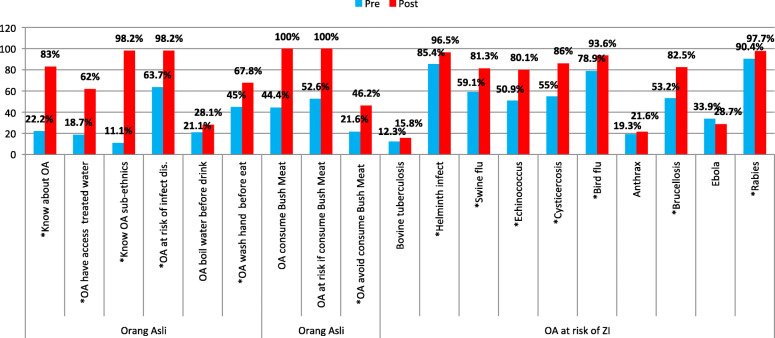
Knowledge relating to *Orang Asli*

Figure 4 shows the knowledge of the participants concerning *Orang Asli*. In general, there is an improvement in the participants’ knowledge concerning *Orang Asli* post workshop. The increase in the knowledge concerning *Orang Asli* (p < 0.001), their access to treated water (p < 0.035), different ethnicities (p < 0.001), risk of infectious diseases (p < 0.001), habits of washing hands before eating (< 0.001), the habit of consuming bush meat (p < 0.001) were statistically significant. The increase in the knowledge for the risk of rabies (< 0.001), brucellosis (< 0.001), bird flu (< 0.001), cysticercosis (p < 0.001), echinococcus (p < 0.001), swine flu and helminth infections (< 0.001) were also statistically significant.

**Fig. 5:**
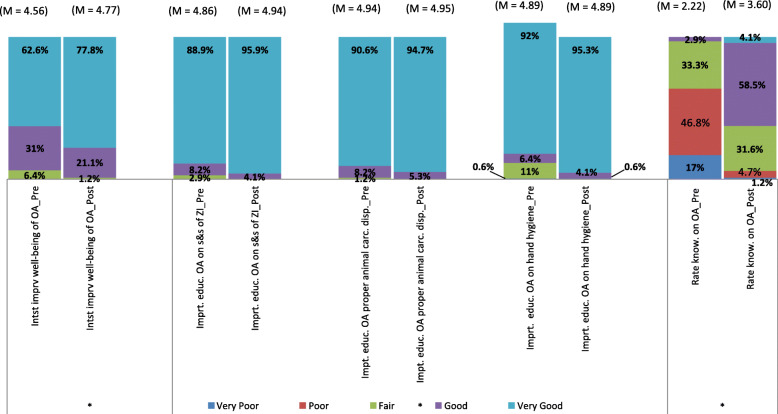
Self-rated knowledge concerning *Orang Asli*

As shown in Fig. 5, the increase in self-rated knowledge concerning *Orang Asli* (p < 0.001), importance of educating the *Orang Asli* to wash hands (< 0.001), proper disposal of animal carcasses (< 0.001), signs and symptoms of zoonotic infections (< 0.001) and the interest in involving in activities for the improvement of the *Orang Asli* (p = 0.046) were statistically significant.

Table 2 shows the increase in the mean knowledge score of the participants was statistically significant (p < 0.001) post workshop. Repeated measures general linear model was conducted to compare the effects of gender and students’ discipline on *Orang Asli* knowledge; however no significant association was found.

**Table 2:** Mean difference in the knowledge on *Orang Asli*

Variable - ***Orang Asli*** knowledge	Mean	Paired t test	Significance
Pre Post	8.38 13.67	− 22.865	< 0.001

Repeated measures ANOVA was conducted to compare the effects of different student courses with One Health and *Orang Asli* knowledge. A repeated measures ANOVA with a Greenhouse-Geisser correction was done as shown in Table 3, the mean scores of knowledge of One Health F (1, 166) = 127.198, p < 0.001) and *Orang Asli* F (1, 166) = 214.757, p < 0.001) differed statistically significantly between the two time points. The test revealed that the score differences for knowledge on One Health (mean difference = 1.796, p < 0.001) and *Orang Asli* (mean difference = 4.940, p < 0.001) were statistically significant. Repeated measures ANOVA showed a significant difference in the *Orang Asli* knowledge between the students in different courses F (4,166) = 3.734, p-0.006. Post hoc analysis showed that knowledge relating to *Orang Asli* scores of students enrolled Public Health course was higher than students from allied health courses (p = 0.01). The difference in the One Health knowledge scores between the students of different courses was not significant F (4,166) = 0.998, p = 0.410.

**Table 3:** Repeated measures ANOVA

Comparison pairs	Mean difference (95%CI)	Significance
**Knowledge on OA**		
PH > Allied Health	2.05 (0.31–3.80)	0.010

## Discussion

Overall, the significant increase in the knowledge relating to One Health and the *Orang Asli* among the participants in these workshops demonstrates evidence that field training in a multidisciplinary environment is effective in the introduction of One Health in all disciplines. This is important, considering at present especially in the public health sector where isolated silo thinking remains a challenge and always persists [22]. This improvement is not altogether unexpected, since these approaches have been proven to be effective in studies which have shown that interactive workshop and field activities improve trainees’ knowledge [23–25]. The interdisciplinary education improves deep disciplinary knowledge, problem-solving skills and self-directed learning habits [26, 27].

In this study, self-rated knowledge on One Health in all disciplines increased significantly showing that majority of the students benefited from these workshops by understanding in depth concerning One Health by fostering interprofessional teaching and learning. This is the key factor in promoting successful collaborative practise using interprofessional education, as suggested by previous studies [28, 29]. It also supports the notion that integrated training platform is crucial in building personal relationships founded on respect and being comfortable working outside the boundaries of one’s discipline to build an effective One Health team that cross species and systems.

Leaning first-hand through field training elicits positive affective response among the trainees and this kind of trainings have been applied extensively in different disciplines [30–32]. Through field training/fieldwork, social capital is developed through group dynamics and barriers are broken down between facilitators, students, and communities. Similar to previous studies, these positive responses from the students in the present study resulted in higher levels of motivation and enhanced learning process [30, 31]. Visit to the *Orang Asli* communities and natural habitat of the wildlife enabled the students to link the risk factors related to the lifestyles of the *Orang Asli* and the risks they are exposed to in the transmission of the zoonotic diseases either directly from animals by bushmeat consumption or by indirect contact. These results are in agreement with previous studies [33, 34]. During the field training too, the students were able to appreciate human animal conflicts and the increase in contact and the resultant risk to zoonotic diseases because of deforestation besides the risks related to the lifestyle of *Orang Asli* communities. This enabled the students to appreciate the efforts needed from all parties, including politicians and decision makers to educate and create awareness on the importance of education among *Orang Asli* communities [3].

Trainees from the medicine and veterinary medicine fared better post workshop as compared with allied health students. This is because there are elements of One Health in relation to agent, host and environment education, which is embedded in the medical and veterinary education curriculum.

### Strength and limitations

The strength of this study is that interdisciplinary education was conducted in real-life situation using experienced multidisciplinary facilitators leading by example of One Health make up and not in traditional classroom settings. There are several limitations to the study. The main limitation was the lack of comparison of the outcome between classroom settings and field training within the same group of trainees. Another limitation of the study is the time period the participants actually spent in an *Orang Asli* village was relatively short due to the restriction of regulations.

### Implications of the study

This study showed that even brief field experience involving multidisciplinary students can produce positive impact in the field of One Health. The approaches and topic of discussions used in this study can be used to develop a module to train future One Health workforce in combating health issues among the *Orang Asli* in larger scale. The findings of this study will be shared with other institutions to encourage field training using the One Health concept. The investigators recommend larger scale studies encompassing longer periods of training be conducted in other *Orang Asli* communities using students from a wider range of disciplines to measure the effectiveness of this form of training.

## Conclusions

This study showed a significant increase in various aspects of knowledge and interest concerning One Health and the *Orang Asli*. Greater emphasis on field training can provide university students with greater levels of preparedness for the future in combating zoonotic diseases. Competency and integrated based education as provided in this study will provide greater reliability of appropriate performance of future One Health team in the field. In order to develop a novel solution for One Health training requires a deep understanding of the human, animal and ecosystem interface.

## Data Availability

All data generated or analysed during this study are included as supplementary information files.
